# Expert opinion on facilitating intrafamily communication in rare diseases—Lessons from Fabry disease

**DOI:** 10.1016/j.gimo.2025.103481

**Published:** 2025-12-02

**Authors:** Dominique P. Germain, Fatma Al-Jasmi, Gheona Altarescu, Olga Azevedo, Fellype C. Barreto, Alessandro P. Burlina, Fatih Ezgü, Dawn A. Laney, Ales Linhart, Manish Maski, Sergey Moiseev, Dau-Ming Niu, Kotaro Nochioka, Yan Ouyang, Huseyin Onay, Mary Pavlou, Nicholas Pachter, Juan Politei, Sunbul Rawda, Richard P. Steeds, Antonino Tuttolomondo, Wen-Chung Yu, Michael L. West, Kenneth I. Berger, Irina Maksimova

**Affiliations:** 1 Division of Medical Genetics, University of Versailles, Montigny, France; 2 Referral Center for Fabry Disease and Lysosomal Diseases, MetabERN, APHP University Paris Saclay, Garches, France; 3 2nd Department of Internal Medicine, Cardiology and Angiology, General University Hospital and 1st Faculty of Medicine of Charles University, Prague, Czech Republic; 4 College of Medicine and Health Sciences, United Arab Emirates University, Al-Ain, United Arab Emirates; 5 Hebrew University Hadassah School of Medicine, Shaare Zedek Medical Center, Jerusalem, Israel; 6 Reference Center of Lysosomal Storage Disorders, Hospital Senhora da Oliveira, Guimaraes, Portugal; 7 Service of Nephrology, Department of Internal Medicine, Federal University of Paraná, Curitiba, Brazil; 8 Neurology Unit, St Bassiano Hospital, Bassano del Grappa, Italy; 9 Gazi University Faculty of Medicine, Ankara, Türkiye; 10 Emory University School of Medicine, Atlanta, GA, USA; 11 Sanofi, Cambridge, MA; 12 Tareev Clinic of Internal Diseases, Sechenov First Moscow State Medical University, Moscow, Russia; 13 Institute of Clinical Medicine, National Yang-Ming University, Taipei, Taiwan; Department of Pediatrics, Taipei Veterans General Hospital, Taipei, Taiwan; 14 Department of Cardiovascular Medicine, Tohoku University Graduate School of Medicine, Sendai, Japan; 15 Department of Nephrology, Institute of Nephrology, Ruijin Hospital, The Medical School of Shanghai Jiao Tong University, Shanghai, China; 16 Multigen Genetic Diseases Diagnosis Center, Izmir, Türkiye; 17 Fabry International Network, Greece; 18 The University of Western Australia, Perth, Australia; Genetic Health WA, King Edward Memorial Hospital, Perth, Australia; 19 Neurology Department, SPINE FUNDATION, Buenos Aires, Argentina; 20 University Institute of Health Sciences, H.A. Barceló Foundation, Buenos Aires, Argentina; 21 Genetic Unit, Pediatric Department, Qatif Central Hospital, Qatif, Saudi Arabia; 22 University Hospitals Queen Elizabeth, Institute of Cardiovascular Sciences, University of Birmingham, Birmingham, United Kingdom; 23 Referrence Centre for Fabry Disease, AOUP “P. Giaccone” University Hospital, Palermo, Italy; 24 Department of Health Promotion, Mother and Child Care, Internal Medicine and Medical Specialties (ProMISE) “G. D’Alessandro,” University of Palermo, Palermo, Italy; 25 Taipei Veterans General Hospital and National Yang Ming Chiao Tung University, Taipei, Taiwan; 26 Division of Nephrology, Department of Medicine, Dalhousie University, Halifax, NS, Canada; 27 Sanofi, Cambridge, MA

## Introduction

Fabry disease (FD, OMIM 301500) is a rare X-linked lysosomal disorder characterized by remarkable heterogeneity in its manifestations and age of onset, leading to significant diagnostic delays.^1^^,^^2^ In FD, pathogenic variants of the *GLA* gene lead to the functional deficiency of α-galactosidase A, causing progressive accumulation of glycosphingolipids, primarily globotriaosylceramide (Gb3) and its deacylated form globotriaosylsphingosine (lyso-Gb3), in various tissues/organs, plasma, and urine.^1^ FD can manifest as “classic” early-onset or “later-onset” forms in both males and females. The classic form typically appears in childhood or adolescence and is marked by early symptoms, including neuropathic pain, gastrointestinal disturbances, hypohidrosis, heat and cold intolerance, *cornea verticillata*, tinnitus, hearing loss, and angiokeratomas. In adulthood severe cardiac, renal and cerebrovascular complications reduce quality of life and life expectancy in the absence of disease specific and symptomatic treatments. The later-onset form appears in adulthood and predominantly involves the heart and more occasionally the kidneys, presenting with signs and symptoms such as hypertrophic cardiomyopathy, heart failure, albuminuria, and proteinuria.^1^^,^^3^ This variable presentation often results in a prolonged diagnostic journey, sometimes taking decades to reach a correct diagnosis.^3^^,^^4^

The multisystemic nature of FD contributes to its diagnostic challenges, particularly in females who may experience symptoms as severe as males or a milder phenotype in relation with their X chromosome inactivation profile.^5^ The severity and progression of these manifestations increases with age, contributing to poor quality of life (QoL) and shortened lifespan, and the most significant contributors are heart failure, cardiac arrhythmias, stroke, end-stage kidney disease, and pain.^1^^,^^4^ Additionally, stigma may also severely affect quality of life.

The incidence of classic FD in males was generally estimated at 1 in 50,000.^6^ However, newborn screening (NBS) studies indicate that the rates vary with geographical region and the definition of FD (ie, also considering later-onset FD).^7^, ^8^, ^9^, ^10^ In NBS, individuals carrying variants of uncertain significance in *GLA* are often detected. In such cases, or when it is unclear whether the individual will develop FD, measuring plasma lyso-Gb3 levels can be helpful in assessing the potential risk of future disease development.^11^ For early diagnosis, screening of high-risk population and family screening are essential. The prevalence of certain pathogenic alleles within specific communities can often be traced back to their founding individuals, illustrating the founder effect. This phenomenon highlights at-risk communities, as observed in Taiwan and other South Asian countries, Nova Scotia (Canada), and Northern Portugal.^8^^,^^12^, ^13^, ^14^

For accurate diagnosis of rare diseases, development and analysis of family pedigree is vital; this also helps in confirming inheritance patterns and identifying potentially affected relatives. Thus, after a patient is diagnosed with FD, clinicians or geneticists should construct a detailed pedigree (including at least three generations) in a standard format, which should be regularly reassessed. This enables easy visualization of the X-linked inheritance pattern and symptom clustering, which aids in the interpretation of clinical phenotyping.^15^ Data privacy regulations should be carefully followed during collection and storage of genetic pedigree information. In agreement with chapter 14 of the European Artificial Intelligence Act, patients should be informed when artificial intelligence might be involved in the processing of their data.

Cascade genetic testing, also called family screening, has emerged as a powerful tool to break the cycle of delayed diagnosis. Systematically identifying and offering testing to individuals at risk for FD enables earlier identification and intervention for affected family members.^2^ Studies have reported that the identification of a proband results on average in the identification of 5 additional affected family members. This underscores the critical importance of effective family screening programs in identifying undiagnosed individuals and providing updated guidelines for FD management.^2^^,^^4^ However, one of the most significant barriers to effective family screening is the lack of appropriate intrafamily communication.^16^ This commentary aims to explore the challenges in building and supporting effective family communication in FD and propose strategies to overcome these barriers, drawing from expert opinions (through previously conducted global expert meetings and surveys)^16^ and recent research in the field.

### Barriers to effective intrafamily communication and strategies to overcome them

#### Knowledge and awareness barriers

##### Barrier

Limited disease understanding and access to educational resources significantly impedes effective family communication for patients with FD.^2^^,^^16^ Probands often struggle to explain the importance of genetic testing to their family members. Silent disease progression and variable disease severity can lead to underestimated importance of testing. Social isolation may occur in families of patients with classic FD and further compounds these challenges.

##### Strategy

It may be crucial to provide simple, accessible educational materials about FD and its inheritance patterns, including audio and video resources for individuals with visual or reading difficulties. Family discussions should be facilitated using standardized questions, and families should also be connected with patient advocacy groups (PAGs) for additional support and resources.^16^ International patient organizations, such as the Fabry International Network (FIN) (https://www.fabrynetwork.org/), facilitate global knowledge sharing and community building among patients, families, and health care providers (HCPs). They bridge gaps through educational events and resources while globally connecting patients for peer support and advocacy. These organizations along with local support groups and online communities provide essential platforms for the patients to share experiences and resources, strengthening coping mechanisms and fostering a sense of community. Annual meetings and genetic counseling services are pivotal for patient support and early diagnosis across different health care systems.^2^

#### Psychological and emotional barriers

##### Barrier

Mental health challenges and emotional factors significantly affect communication in the families of patients with FD.^17^ Depression and anxiety are documented symptoms of FD that can prevent open discussion about FD, particularly when combined with feelings of guilt, shame, and denial. The social stigma associated with a genetic condition creates an additional barrier as a diagnosis could lead to disparity in education, employment, health care, and QoL, as well as psychological distress. Furthermore, patients may encounter various forms of social stigma within the health care setting, echoing experiences common among those living with chronic illnesses.^18^^,^^19^

##### Strategy

Psychological support should be integrated into the care team by providing counseling resources for both patients and family members to address emotional challenges. Creation of safe spaces through support groups and peer networks can also help both patients and their families in the navigation of the social stigma and psychological aspects of FD.^17^

#### Family dynamics and social barriers

##### Barrier

Complex or strained family relationships, fragmented family structures, and social factors can hinder or complicate communication about FD. Geographical dispersion and financial barriers to accessing health care, diagnosis, and treatment further limit intrafamily communication. Cultural and societal factors may also affect attitude toward genetic testing and disclosure.^2^^,^^20^

##### Strategy

A “family champion” should be identified to lead communication efforts. Geographical barriers can be overcome by using technology for virtual family meetings and information sharing. Finally, culturally sensitive guidance and support should be provided for navigating family dynamics and effective communication across diverse family structures.

#### Health care system barriers

##### Barrier

Limitations within health care systems can impede effective communication about FD,^2^ eg, the lack of standardized protocols for family communication in rare diseases, variable availability of genetic counseling resources across regions (Supplemental Table 1), and limited expertise regarding FD management among nonspecialist HCPs. Not every patient may have full access to health care, diagnosis and even basic therapy.

##### Strategy

Multidisciplinary teams including FD experts should be established.^4^ Telemedicine or artificial intelligence solutions should be implemented to bridge gaps in specialized care, especially for geographically dispersed families.^21^^,^^22^ To ensure a consistent approach among HCPs, standardized communication protocols for rare diseases should be developed and disseminated.

#### Communication barriers and strategies

##### Barrier

Ineffective communication techniques can hinder the process of information sharing and disease understanding in the families of patients with FD.^16^^,^^23^ Patients and HCPs may struggle with conveying complex genetic information, and the timing and approach to sensitive discussions may be suboptimal. Lack of follow-up and ongoing support in the communication process can also impede effective family dialog.

##### Strategy

HCPs should be trained in effective communication techniques for genetic counseling.^23^ Discussion with patients about strategic timing, such as during holidays when families gather, and techniques for opening family discussions can improve receptivity to information. Continuous support and multiple opportunities should be provided to family members for clarification of queries; this will ensure active engagement and understanding throughout FD journey.

### Implementing effective communication strategies

A comprehensive approach is needed to overcome intrafamily communication barriers and facilitate better intrafamily discussion about FD. The following strategies, based on expert consensus, can significantly improve the process of family screening and communication in FD^16^^,^^17^^,^^23^:

#### Establishing multidisciplinary team

The team should include cardiologists, nephrologists, neurologists, pain specialists, pediatricians, geneticists, genetic counselors, psychologists, nurses, and social workers with FD expertise. In an ideal patient-centered care model, each specialist would conduct thorough evaluations and then collaborate to develop a multidisciplinary care plan for the patient. Often the core of this team is a specialist in FD (“Fabryologist”), who can provide care coordination and centralized communication. This comprehensive approach will ensure effective management of all disease aspects, improving patient outcomes.

#### Personalized approach

Tailor communication strategies to each family’s unique dynamics, cultural background, and overall level of literacy and understanding.

#### Staged information delivery

Provide information in manageable chunks over multiple sessions to avoid overwhelming family members.

#### Utilize visual aids

Use diagrams, family trees, and other visual tools to explain inheritance patterns, disease impact, and the importance of family screening.^15^

#### Leverage technology

Use secure digital platforms for sharing information, scheduling follow-ups, and facilitating ongoing communication between HCPs and families.

#### Empower patient advocates

Support and train patients who are willing to become advocates within their families, equipping them with the knowledge and resources to facilitate discussions.

#### Create supportive environments

Collaborate with PAGs to provide emotional support, peer connections, and additional resources for families navigating FD.

#### Regular follow-up

Implement a system for regular check-ins with families to address new questions, provide updates on advances in FD management, and reinforce the importance of ongoing screening.

#### Identify key individuals

Designate a family member who leads communication efforts and promotes engagement within the family.

#### Anticipate emotional impact

Before discussing significant information, assess the patient’s emotional state; if the patient is overwhelmed, then allow time for processing. Periodic visits should be provided to the patients to psychologist or psychiatrist as needed. Address potential feelings of depression, anxiety, and suffering by explaining available support and effective treatments.^17^

#### Optimal timing

Choose appropriate moments for discussion, such as during family gatherings, to maximize engagement of family members and their receptivity to the information.

### Conclusion

Effective intrafamily communication is crucial for successful family screening, early diagnosis, and improved management of many genetic diseases. HCPs can significantly enhance the care and outcomes for patients and their families by addressing the multifaceted barriers to communication and by implementing comprehensive strategies.

The key to this approach is the establishment of multidisciplinary care teams, provision of ongoing education and support for both patients and families, and utilization of PAGs in accordance with local rules, guidelines, and laws, where applicable. Additionally, leveraging technology for telemedicine solutions and digital communication can help overcome geographical and logistical barriers.^22^

The consensus among experts is that improving disease awareness and supporting patients through PAGs can empower both patients and their families, thereby encouraging them to undergo early screening. Moreover, non-geneticists should be trained in effective communication strategies, and cascade genetic testing should be promoted. Establishing a multidisciplinary team of specialists and facilitating teleconsultations can support patients, particularly in remote areas by enabling early identification of affected relatives.

The commentary reflects experiences from countries with established health care and educational infrastructures; however, in regions with more limited access to such resources, diagnostic pathways and disease management may differ.

To further enhance family communication, trust building, and continuous education for both patients and family members are essential. Moreover, rare disease management can be improved by the implementation of specific measures, such as dedicating protected time for counseling, connecting patients with support groups, and enhancing accessibility to clinical psychologists and genetic counselors. The authors agree that these strategies have the potential to significantly improve clinical practice, enhancing care for patients and their families.

Along with the evolution of our understanding of FD and other rare genetic disorders, we should continuously refine our approaches toward family communication and screening. Thus, open dialog, comprehensive support, and continuous refinement of communication strategies should be focused upon for earlier diagnosis and better management and QoL for patients with genetic diseases.

## Data Availability

Data are available from the corresponding author upon request.

## Conflict of Interest

D.P. Germain: Honoraria and consulting fees from Chiesi, Idorsia, Pfizer, Sanofi, and Takeda. F. Al-Jasmi: Speaker honorarium, travel grant, research grant, and/or fees for participation in advisory boards from Sanofi Genzyme, Chiesi, and Takeda. G. Altarescu: Speaker honoraria from Amicus Therapeutics, Sanofi, and Takeda. O. Azevedo: Research grants and/or travel and accommodation support for congresses from Takeda, Amicus, Sanofi, and Chiesi. F.C. Barreto: Speaker honoraria from Sanofi. A.P. Burlina: Speaker honoraria and fees for participation in advisory boards from Chiesi and Sanofi. F. Ezgü: Principal investigator in clinical studies and a member of some advisory boards sponsored by Sanofi. D. Laney: Honoraria from Amicus Therapeutics, Sanofi, and Takeda; consulting fees from Sanofi, Chiesi, and Takeda; and a cofounder of ThinkGenetic, Inc. A. Linhart: Research grants, consulting fees, speaker fees, and travel support from Amicus Therapeutics, Protalix, Chiesi, Sanofi, and Takeda. S. Moiseev: Travel grants and/or fees for participation in advisory boards from Sanofi and Takeda. K. Nochioka: Speaker honoraria from Sanofi, Amicus Therapeutics, Sumitomo Pharma, and Takeda. Y. Ouyang: An investigator in clinical studies and a member of some advisory boards sponsored by Sanofi and Takeda. H. Onay: Travel grants and/or fees for participation in advisory boards from Sanofi. M. Pavlou: Grants from Chiesi, Sanofi, Takeda, and Amicus Therapeutics; payment for expert testimony from Sanofi and Takeda; and a leadership or fiduciary role in the Fabry International Network and other boards, societies, committees, or advocacy groups, unpaid, from the Fabry International Network and/or the Greek Lysosomal Association. N. Pachter: Travel grants and/or fees for participation in advisory boards from Sanofi. J. Politei: Honoraria from Amicus Therapeutics and Sanofi and consulting fees from Sanofi, Gador, Biosidus, Pint, and Takeda. R.P. Steeds: Honoraria from Amicus Therapeutics, Sanofi, and Takeda and research funding from Sanofi and Takeda. W.-C. Yu: Speaker honoraria from Sanofi and Takeda. M.L. West: Research grants, consulting fees, speaker fees, and travel support from Amicus Therapeutics, Protalix, Sanofi, and Takeda. I. Maksimova and K.I. Berger: Sanofi employees and may hold shares and/or stock options in the company. S. Rawda, A. Tuttolomondo, D.-M. Niu, and M. Maski: declare no conflicts of interest.
