# Supplementary material for: Expert opinion on facilitating intrafamily communication in rare diseases—Lessons from Fabry disease
Source: Genet Med Open. 2025 Dec 2;4:103481. doi: 10.1016/j.gimo.2025.103481 (PMC12809085; doi:10.1016/j.gimo.2025.103481)
Supplement: Supplemental Table 1 [file mmc1.docx]

**Supplementary Table:**

**Supplementary Table 1. Global variability in genetic counseling resources for FD**

| \| **Region/country** \| \| --- \| | \| **Practices** \| \| --- \| | \| **Challenges, if any** \| \| --- \| |
| --- | --- | --- | --- | --- | --- |
| **France** | - Genetic counseling performed by medical geneticists and genetic counselors - Genetic/biochemical tests reimbursed - Labeled referral centers for rare diseases (e.g., [www.centre-geneo.com](http://www.centre-geneo.com)) | - Adequate resources available; newly diagnosed patients are referred to genetic counseling |
| **United States** | - Follows guidelines for genetic counseling | - Adequate resources available; newly diagnosed patients are referred to genetic counseling |
| **Japan** | - Limited number of genetic counselors, primarily focused on cancer | - Few counselors are experienced in rare diseases, such as FD, hindering family counseling efforts |
| **LATAM** | - General practitioners take responsibility for providing genetic advice and guiding family screening | - Most hospitals lack specific genetic counseling services in LATAM regions |
| **Turkey** | - Genetic counseling performed by medical geneticists and specialized physicians - Reimbursement for genetic/biochemical tests in government hospitals - Multidisciplinary follow-up care led by metabolic specialists | - Family screening is typically limited to first-degree relatives, with low awareness among some medical professionals in Turkey |
| **Portugal** | - Genetic counseling provided by medical geneticists | - No dedicated genetic counselors; physicians specialized in genetics manage all types of counseling |
| **Italy** | - Genetic counseling provided by medical geneticists | - Medical geneticists are present in accredited centers for genetic diseases |
| **China** | - Structured genetic counseling process in major cities | - Limited access in rural areas; patients living in remote areas rely on telemedicine networks |
| \| **Greece** \| \| --- \| | - General practitioners manage genetic advice and family screening | - Underdeveloped genetic counseling services |
| \| **Western Australia** \| \| --- \| | - Multidisciplinary FD clinics with geneticists and genetic counselors | - Emphasis on cascade testing and family communication, aided through pamphlets, letters, and follow-ups with genetic counselors and therapy nurses |
| **Czech Republic** | - Genetic counseling and subsequent gene sequencing are only available upon request from a clinical geneticist | - A shortage of geneticists, leading to significant delays in accessing test results. While DBS testing has mitigated this issue for FD, this approach is not a long-term solution and complicates the testing process |

DBS, dried blood spot; FD, Fabry disease; LATAM, Latin America.
